# Host-guest complexation of cucurbit[8]uril with two enantiomers

**DOI:** 10.1038/srep44717

**Published:** 2017-03-16

**Authors:** Zhong-Zheng Gao, Rui-Lian Lin, Dong Bai, Zhu Tao, Jing-Xin Liu, Xin Xiao

**Affiliations:** 1 Key Laboratory of Macrocyclic and Supramolecular Chemistry of Guizhou Province, Guizhou University, Guiyang 550025, P. R. China; 2 College of Chemistry and Chemical Engineering, Anhui University of Technology, Maanshan 243002, P. R. China

## Abstract

Host-guest complexation of cucurbit[8]uril (Q[8]) with two enantiomers, D-3-(2-naphthyl)-alanine (D-NA) and L-3-(2-naphthyl)-alanine (L-NA), has been fully investigated. Experimental data indicate that double guests reside within the cavity of Q[8] in both aqueous solution and solid state, generating highly stable homoternary complexes D-NA_2_@Q[8] and L-NA_2_@Q[8].

The recognition and sensing of amino acids, peptides and proteins in aqueous solution by artificial receptors has attracted much interest in supramolecular host-guest chemistry and pharmaceutical science in the recent past^1^,^2^,^3^,^4^,^5^. The interest is stimulated by its potential applications in diverse fields such as drug delivery, nutritional analysis and disease diagnosis. Various examples of the use of artificial receptors for amino acid (or peptide/protein) recognition have been reported^6^,^7^,^8^,^9^,^10^,^11^,^12^. Cucurbit[*n*]urils (*n* = 5–8, 10, abbreviated as Q[*n*], Fig. 1a) are a family of unique macrocyclic cavitands possessing two identical carbonyl-laced portals and a rigid hydrophobic cavity, which can selectively accommodate and interact with various organic molecules^13^,^14^,^15^,^16^,^17^. In the past decade, Q[*n*]s have been exploited for binding amino acids, peptides and proteins^18^,^19^,^20^,^21^,^22^,^23^,^24^,^25^,^26^,^27^,^28^,^29^,^30^,^31^,^32^,^33^,^34^,^35^. For example, Urbach and co-workers studied the combining power of Q[7] to a number of amino acids, peptides and proteins, and found that the Q[7] prefer to bind guests containing an N-teminal aromatic residue^18^,^19^,^20^,^21^. Kim group systematically studied the binding properties of Q[7] to a series of amino acids in both solution and the gas phase^28^. Scherman *et al*. reported heteroternary and homoternary complexes between Q[8] and peptides with aromatic residues^30^.

It is well known that all alpha amino acids but glycine usually exist in two enantiomers (L- or D-amino acid). To the best of our knowledge, however, the detection and recognition of specific enantiomeric amino acids by Q[*n*]s have never been reported. Previous investigation revealed that the host Q[8] is large enough to accommodate two phenyl, naphthyl or other aromatic groups simultaneously through host-stabilized charge-transfer interactions^14^,^36^,^37^,^38^. This observation prompted us to explore the possibility of the formation of homoternary complexes between Q[8] and enantiomeric amino acid containing naphthyl residue, D-3-(2-naphthyl)-alanine and L-3-(2-naphthyl)-alanine (abbreviated as D-NA and L-NA, respectively, Fig. 1b). In the present work, we studied the host-guest complexation of Q[8] with D-NA and L-NA in aqueous solution by NMR, UV and fluorescence spectroscopy, MS and isothermal titration calorimetry (ITC), and in the solid state by X-ray crystallography.

## Results and Discussion

### Binding Behaviors in Aqueous Solution

**Figure 2:**
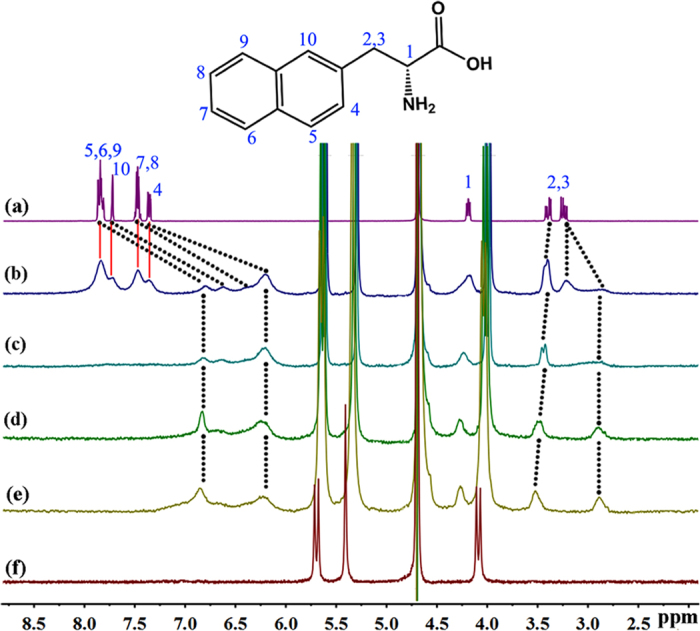
^1^H NMR spectra of 5.0 mM D-NA (**a**), D-NA and Q[8] in the ratio of 0.6 (**b**), 1.1 (**c**), 2.2 (**d**), 3.1 (**e**), and Q[8] (**f**) in D_2_O at 293 K.

The ^1^H NMR spectroscopy measurements indicate that both D-NA and L-NA form host-guest inclusion complex with Q[8] host. Given that the changes induced by Q[8] host in the ^1^H NMR spectra of guests D-NA (Fig. 2) and L-NA (Figure S2) are similar, guest D-NA is taken as a representative to depict their binding interactions. In the presence of small amount of the Q[8] host (Fig. 2b), the signals of both free and complexed guests are simultaneously observed and are very broad, indicating slow exchange of free and complexed guests on the NMR time scale. All guest aromatic protons move upfield considerably, revealing deep insertion of the naphthyl group inside the cavity. On the other hand, the proton H^1^ and one of the CH_2_ protons of D-NA move downfield slightly, which indicates that they are located outside the cavity. At a 2:1 ratio of D-NA to Q[8], the aromatic peaks are completely shifted upfield. These observations suggest that the naphthyl moiety of the D-NA guest was encapsulated into the cavity of the Q[8] host.

**Figure 3:**
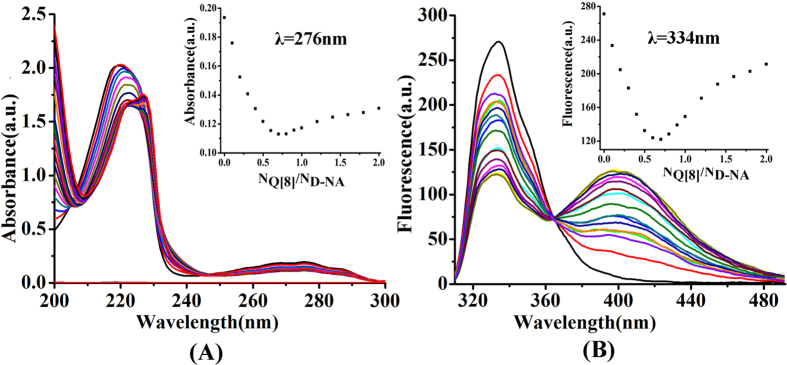
UV spectra of D-NA (2.0 × 10^−5^) (**A**) and fluorescence spectra of D-NA (2.0 × 10^−5^) (**B**) with increasing concentration (0.0, 0.1, 0.2, 0.3, 0.4, 0.5, 0.6, 0.7, 0.8, 0.9, 1.0, 1.2, 1.4, 1.6, 1.8, 2.0 equiv) of Q[8]. The inset shows the formation of a 1:2 host-guest complex.

To better understand the host-guest interaction between Q[8] and both enantiomers in aqueous solution, we carried out UV and fluorescence titration experiments. According to the UV absorption spectroscopic results, Fig. 3(A), upon the gradual addition of Q[8] into D-NA in H_2_O, the absorption underwent a slight bathochromic shift from 220 to 227 nm in addition to a significant decrease in its intensity due to the strong interaction between Q[8] and D-NA. This is actually also true for the case of Q[8] with L-NA (Figure S3).

We also studied the fluorescence properties of both D-NA and L-NA in the presence of Q[8]. As can be seen in Fig. 3(B), the D-NA shows an emission peak at 334 nm in aqueous solution, when the excitation is λ = 274 nm. Successive addition of Q[8] caused decrease in the fluorescence intensity at 334 nm and appearance of a new emission peak at around λ = 410 nm. Moreover, we found that an isobestic point appears at 364 nm. These substantial changes in emission profiles further confirm the strong host-guest interaction between Q[8] and D-NA. When the D-NA is replaced by L-NA, similar fluorescence spectra are also observed (Figure S3).

Their Job’s plots (based on the continuous variation method) clearly show that both UV and fluorescence spectra data of both enantiomers fit well to 1:2 stoichiometry of the host-guest inclusion complexes (Fig. 3, inset). The formation of the homoternary complexes D-NA_2_@Q[8] and L-NA_2_@Q[8] was also established by the MS experiments. Their MALDI-TOF spectra (Figure S4, Supporting Information) gave doubly-charged peak at *m/z* = 880.2700 for the D-NA_2_@Q[8], and *m/z* = 880.2884 for the L-NA_2_@Q[8] (calculated for [2D-NA@Q[8]-2Cl^−^]^2+^/2 and [2L-NA@Q[8]-2Cl^−^]^2+^/2, 880.8958).

### Structural Analysis

**Figure 4:**
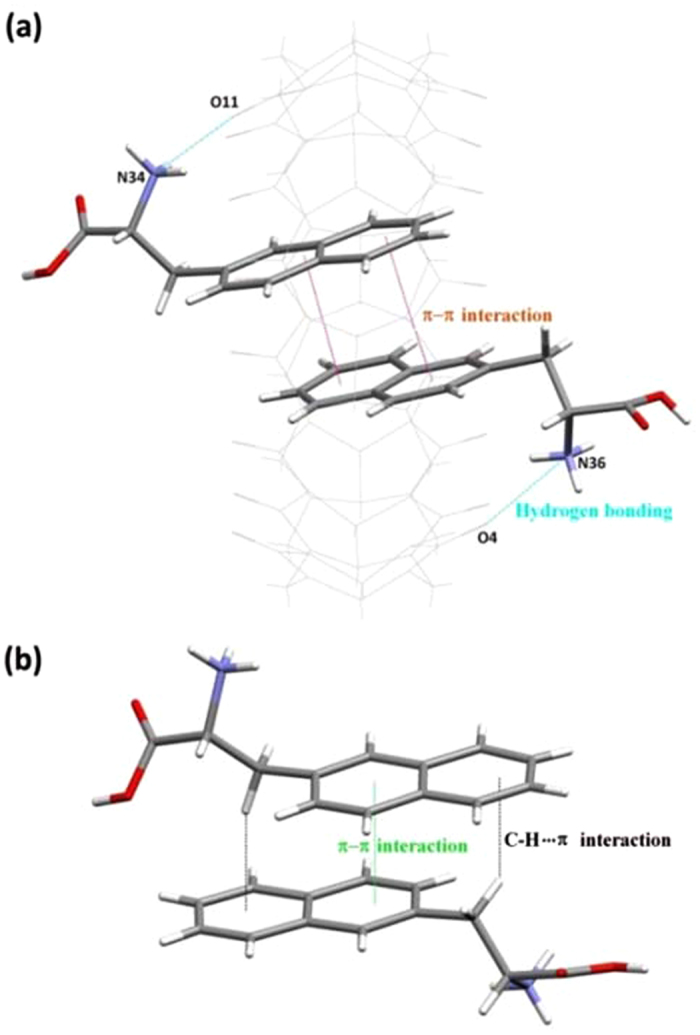
(**a**) X-ray crystal structure of the homoternary complex D-NA_2_@Q[8]. Free D-NA molecules, solvate water molecules and [CdCl_4_]^2−^ anions are omitted for clarity. (**b**) The C–H···π interaction between two neighboring D-NA molecules outside of the Q[8].

X-ray structure analysis provided unequivocal proof of the formation of homoternary complexes between Q[8] and both enantiomers. Crystals of D-NA_2_@Q[8] were grown by slow evaporation of a solution containing the host Q[8] and the guest D-NA under 3.0 M aqueous hydrochloric acid solution in the presence of CdCl_2_. X-ray structural analysis has established that the D-NA_2_@Q[8] crystallize in the monoclinic crystal system, space group *P*2_1_/*c*. As can be seen in Fig. 4, the naphthyl moiety of the D-NA guest was located inside the cavity of the Q[8] host, which is in agreement with what we have observed in the aqueous solution by ^1^H NMR spectroscopy. Obviously, the van der Waals contacts between the naphthyl groups and the inner wall of the Q[8] cavity, together with the electrostatic interactions between the protonated nitrogens in the guests and the carbonyl oxygens at the portals of the Q[8] host, and strong hydrogen-bonding: N(18)–H···O(4) 2.860(5) Å, N(34)–H···O(11) 2.902(3) Å, contribute to the formation of the inclusion complex D-NA_2_@Q[8]. Furthermore, the π···π interactions between two encapsulated D-NA molecules play a critical role in the formation of this host-guest inclusion complex. Outside of the inclusion complexes, neighboring D-NA molecules contact with each other through not only π···π interaction, but also C–H···π interactions.The synthesis and structure of L-NA_2_@Q[8] (Figure S5, Supporting Information), is similar to that of D-NA_2_@Q[8], except that the D-NA is replaced by the L-NA.

It is should be noted that the homoternary complex D-NA_2_@Q[8] is completely different from the host-stabilized charge-transfer complexes, which Kim group perviously reported^14^. In the homoternary complex D-NA_2_@Q[8], the two encapsulated D-NA molecules are connected together through π···π interactions. In the latter, the encapsulated guests were electron donor and acceptor pair, and the major driving force for the ternary complex formation appears to be strong charge-transfer interaction between the guests^14^.

### Description of ITC

**Figure 5:**
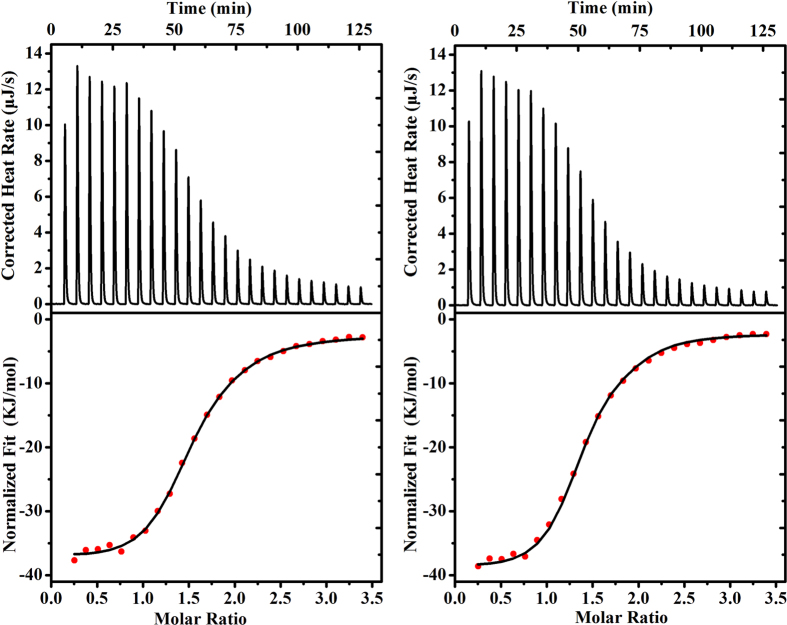
ITC profile of Q[8] with (**a**) D-NA and (**b**) L-NA at 298.15 K.

ITC study (Fig. 5) on the complexation of Q[8] with both D-NA and L-NA affords the thermodynamic parameters (Table S1), and further confirms that the binding stoichiometry of Q[8] to both enantiomers is 1:2. From the Δ*H* and *T*Δ*S* values in the Table S1, it is clear that the formation of both homoternary complexes is enthalpically driven. The observed negative enthalpy change (Δ*H*_1_ = −50.12 ± 2.59 kJ·mol^−1^, Δ*H*_2_ = −6.17 ± 2.56 kJ·mol^−1^ for D-NA_2_@Q[8]; Δ*H*_1_ = −48.97 ± 4.42 kJ·mol^−1^, Δ*H*_2_ = −3.97 ± 4.77 for L-NA_2_@Q[8]) is probably due to the cooperativity of above mentioned four kinds of weak interactions. On the basis of the corresponding experimental results, we also obtained the association constants of *K*a = (6.51 ± 0.19) × 10^11^ M^−2^ and (3.17 ± 0.05) × 10^11^ M^−2^ for Q[8] with D-NA and L-NA, which are much larger than that of Q[8] with tripeptides reported by Urbach^21^. Such a high binding constant suggests the relatively strong host-guest interaction between Q[8] and D-NA or L-NA, indicating the construction of stable homoternary complexes D-NA_2_@Q[8] and L-NA_2_@Q[8] in aqueous solution.

### Conclusion

In summary, we have investigated the host-guest complexation of Q[8] with two enantiomers D-NA and L-NA in both aqueous solution and solid state by using NMR, UV and fluorescence spectroscopy, MS, isothermal titration calorimetry (ITC), and X-ray crystallography. Driven by the cooperativity of electrostatic interactions, multiple C–H···π interactions, and hydrogen-bondings, both D-NA and L-NA can be encapsulated into the cavity of Q[8] to form stable homoternary complexes D-NA_2_@Q[8] and L-NA_2_@Q[8]. This study suggests that Q[8] host may be very useful in dimerisating specific amino acids, peptides and proteins with suitable binding groups.

## Methods

### Materials and methods

3-(2-naphthyl)-D-alanine and 3-(2-naphthyl)-L-alanine were obtained from Aldrich and used as supplied without further purification. Q[8] was prepared according to a literature method^39^,^40^. All the ^1^H NMR spectra were recorded on a Bruker DPX 400 spectrometer in D_2_O. Absorption spectra of the host-guest complexes were recorded on an Aglient 8453 spectrophotometer at room temperature. Fluorescence spectra of the host-guest complexes were performed with a Varian RF-540 fluorescence spectrophotometer. MALDI-TOF mass spectrometry was recorded on a Bruker BIFLEX III ultra-high resolution Fourier transform ion cyclotron resonance (FT-ICR) mass spectrometer with a-cyano-4-hydroxycinnamic acid as matrix.

### Single-crystal X-ray crystallography

Single crystals of D-NA_2_@Q[8] and for L-NA_2_@Q[8] were grown from hydrochloride acid solution by slow evaporation. Diffraction data of both complexes were collected at 273(2) K with a Bruker SMART Apex-II CCD diffractometer using graphite-monochromated Mo-*K*_α_ radiation (λ = 0.71073 Å). Empirical absorption corrections were performed by using the multi-scan program SADABS. Structural solution and full-matrix least-squares refinement based on *F*^*2*^ were performed with the SHELXS-97 and SHELXL-97 program packages, respectively^41^,^42^. Non-hydrogen atoms were treated anisotropically in all cases. All hydrogen atoms were introduced as riding atoms with an isotropic displacement parameter equal to 1.2 times that of the parent atom. Hydrogen atoms were given for all isolated water molecules.

Crystal data for D-NA_2_@Q[8]: [(C_13_H_14_NO_2_)_2_@(C_48_H_48_N_32_O_16_)]·(CdCl_4_^2−^)_2_·(C_13_H_14_NO_2_)_2_·10(H_2_O), *M*_*r*_ = 2882.77, monoclinic, space group *P2*_*1*_*/c, a* = 18.875(2) Å, *b* = 12.5113(16) Å, *c* = 27.418(3) Å, *β* = 112.540(6)°, *V* = 5980.2(12) Å^3^, *Z* = 2*, Dc* = 1.601 g cm^−3^, *F*(000) = 2959, *GOF* = 1.008, *R*_*1*_ = 0.1183 *(I* > *2σ(I)), wR*_*2*_ = 0.3541 (all data).

Crystal data for L-NA_2_@Q[8]: [(C_13_H_14_NO_2_)_2_@(C_48_H_48_N_32_O_16_)]·(CdCl_4_^2−^)_2_·(C_13_H_14_NO_2_)_2_·10(H_2_O), *Mr* = 2882.77, monoclinic, space group *P*2_1_*/c, a* = 18.875(2) Å, *b* = 12.5113(16) Å, *c* = 27.418(3) Å, *β* = 112.540(6)°, *V* = 5980.2(12) Å^3,^ *Z* = 2, *Dc* = 1.601 g cm−3, *F*(000)* = *2959, *GOF* = 1.007, *R*_1_ = 0.1164 (*I* > *2σ(I)*), *wR*_2_ = 0.3555 (all data).

CCDC 1451630 and 1451631 contain the supplementary crystallographic data for this paper. These data can be obtained free of charge from The Cambridge Crystallographic Data Centre via www.ccdc.cam.ac.uk/data_request/cif.

## Preparation of D-NA_2_@Q[8] and L-NA_2_@Q[8]

### Synthesis of the crystal D-NA_2_@Q[8]

To a solution of D-NA (10.8 mg, 0.050 mmol) and CdCl_2_ (3.6 mg, 0.050 mmol) in 3.0 M HCl (2 ml), Q[8] (6.2 mg, 0.005 mmol) was added. The resulting reaction mixture was stirred for 5 min at 50 °C and filtered. Slow solvent evaporation of the filtrate in air over a period of about two weeks provided rhombic colorless crystals of D-NA_2_@Q[8] with the yield of 1.8 mg (20%).

### Synthesis of the crystal L-NA_2_@Q[8]

To a solution of L-NA (10.8 mg, 0.050 mmol) and CdCl_2_ (3.6 mg, 0.050 mmol) in 3.0 M HCl (2 ml), Q[8] (6.2 mg, 0.005 mmol) was added. The resulting reaction mixture was stirred for 5 min at 50 °C and filtered. Slow solvent evaporation of the filtrate in air over a period of about three weeks provided rhombic colorless crystals of L-NA_2_@Q[8] with the yield of 1.7 mg (18%).

### Isothermal titration calorimetry (ITC) experiments

ITC data were obtained on a Nano ITC instrument (TA, USA). Titration were performed with Q[8] concentration of approximately 0.1 mM in the sample cell (1.3 mL), and D-NA or L-NA concentration of approximately 2 mM in the syringe (250 μL). The heat of dilution was corrected by injecting the guest solution into deionized water and subtracting these data from those of the host-guest titration. All titrations were repeated three times. Computer simulations (curve fitting) were performed using the Nano ITC analyze software.

## Additional Information

**How to cite this article:** Gao, Z.-Z. *et al*. Host-guest complexation of cucurbit[8]uril with two enantiomers. *Sci. Rep.* **7**, 44717; doi: 10.1038/srep44717 (2017).

**Publisher's note:** Springer Nature remains neutral with regard to jurisdictional claims in published maps and institutional affiliations.

## Supplementary Material

Supporting Information

## Figures and Tables

**Figure 1:**
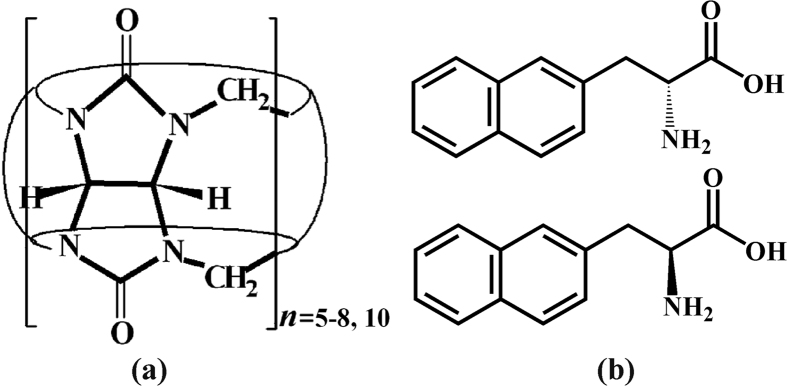
Structures of the Q[*n*] and guests used in this study.
