# Supplementary material for: Host-guest complexation of cucurbit[8]uril with two enantiomers
Source: Sci Rep. 2017 Mar 16;7:44717. doi: 10.1038/srep44717 (PMC5353740; doi:10.1038/srep44717)
Supplement: Supporting Information [file srep44717-s1.doc]

**Supporting Information**

**Host-guest complexation of cucurbit[8]uril with two enantiomers**

Zhong-Zheng Gao,^1^ Rui-Lian Lin,^2^ Dong Bai,^1^ Zhu Tao,^1^Jing-Xin Liu,^2^*and Xin Xiao^1*^

_____________________________________________________________________

*1* Z.-Z. Gao, D. Bai, Z. Tao, X. Xiao

Key Laboratory of Macrocyclic and Supramolecular Chemistry of Guizhou Province, Guizhou University, Guiyang 550025, P. R. China

E-mail: gyhxxiaoxin@163.com

*2* R.-L. Lin, J.-X. Liu

College of Chemistry and Chemical Engineering, Anhui University of Technology, Maanshan 243002, P. R. China

E-mail: jxliu411@163.com

**Table of Contents**

Figure S1. ^1^H-^1^H COSY spectra of D-NA in DMSO................................................................ ..1

Figure S2. ^1^H NMR spectroscopic data of Q[8] with guest L-NA in D_2_O...................................2

Figure S3. UV spectra of L-NA (a) and fluorescence spectra of L-NA (b) with increasing concentration (0.0, 0.1, 0.2, 0.3, 0.4, 0.5, 0.6, 0.7, 0.8, 0.9, 1.0, 1.2, 1.4, 1.6, 1.8, 2.0 equiv) of Q[8].........................................................................................................3

Figure S4. The MALDI-TOF mass spectrum for D-NA_2_@Q[8] and L-NA_2_@Q[8]................... 4

Figure S5. X-ray crystal structure of the homoternary complex L-NA_2_@Q[8]...........................5

Table 1. Complex stability constant (*K*_a_), enthalpy (Δ*H*°), and entropy changes (*T*Δ*S*°) for D-NA_2_@Q[8] and L-NA_2_@Q[8]................................................................................. 6


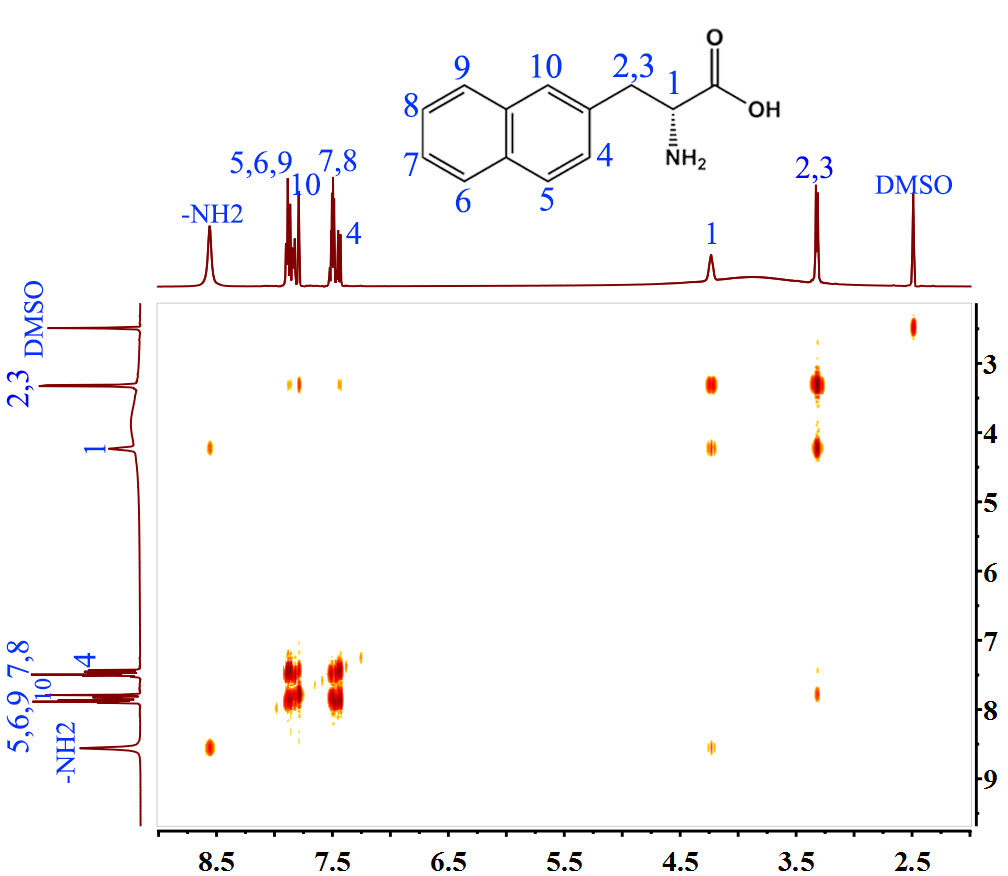


**Figure S1.** ^1^H-^1^H COSY spectra of D-NA in DMSO.


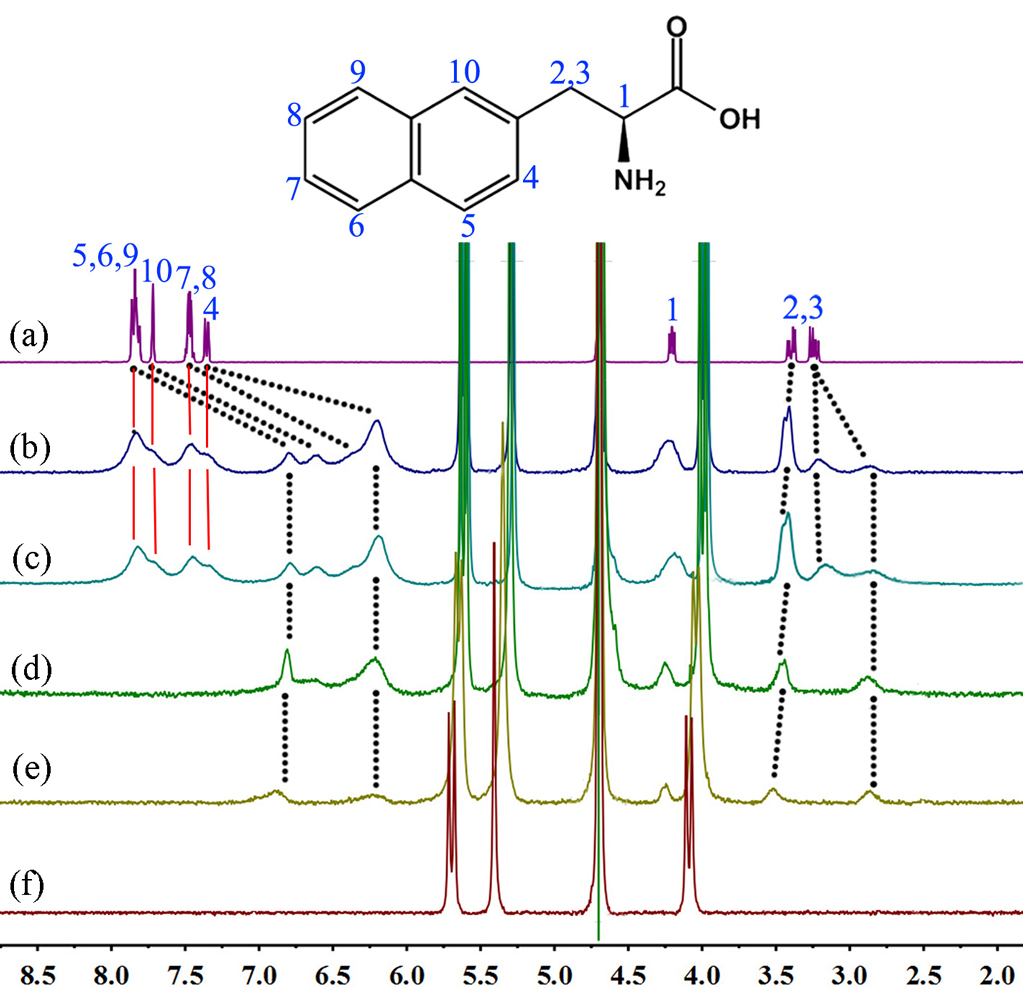


**Figure S2** ^1^H NMR spectra of L-NA (a), L-NA and Q[8] in the ratio of 0.5 (b), 0.9 (c), 1.9 (d) , 2.8 (e), and Q[8] (f) in D_2_O at 293K.


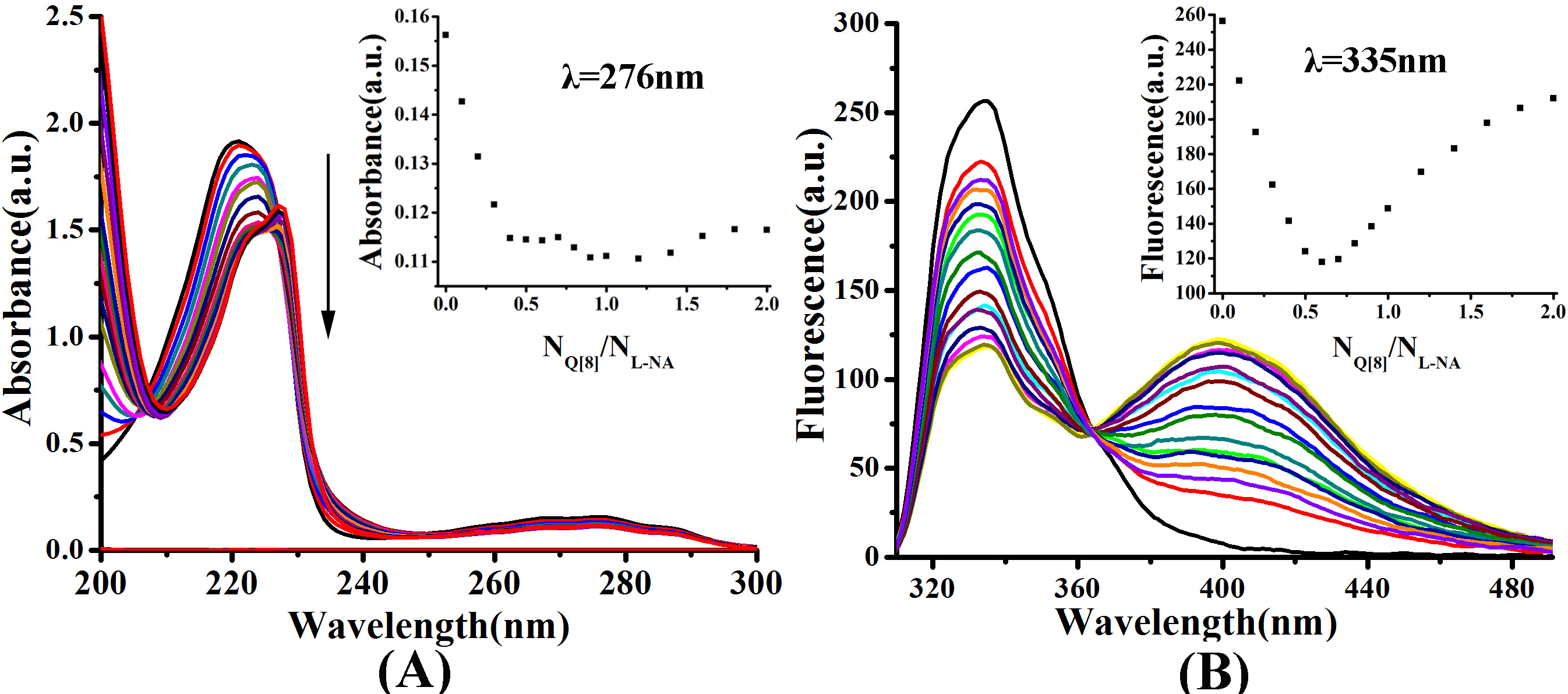


**Figure S3** UV spectra of L-NA (a) and fluorescence spectra of L-NA (b) with increasing concentration (0.0, 0.1, 0.2, 0.3, 0.4, 0.5, 0.6, 0.7, 0.8, 0.9, 1.0, 1.2, 1.4, 1.6, 1.8, 2.0 equiv) of Q[8].

**
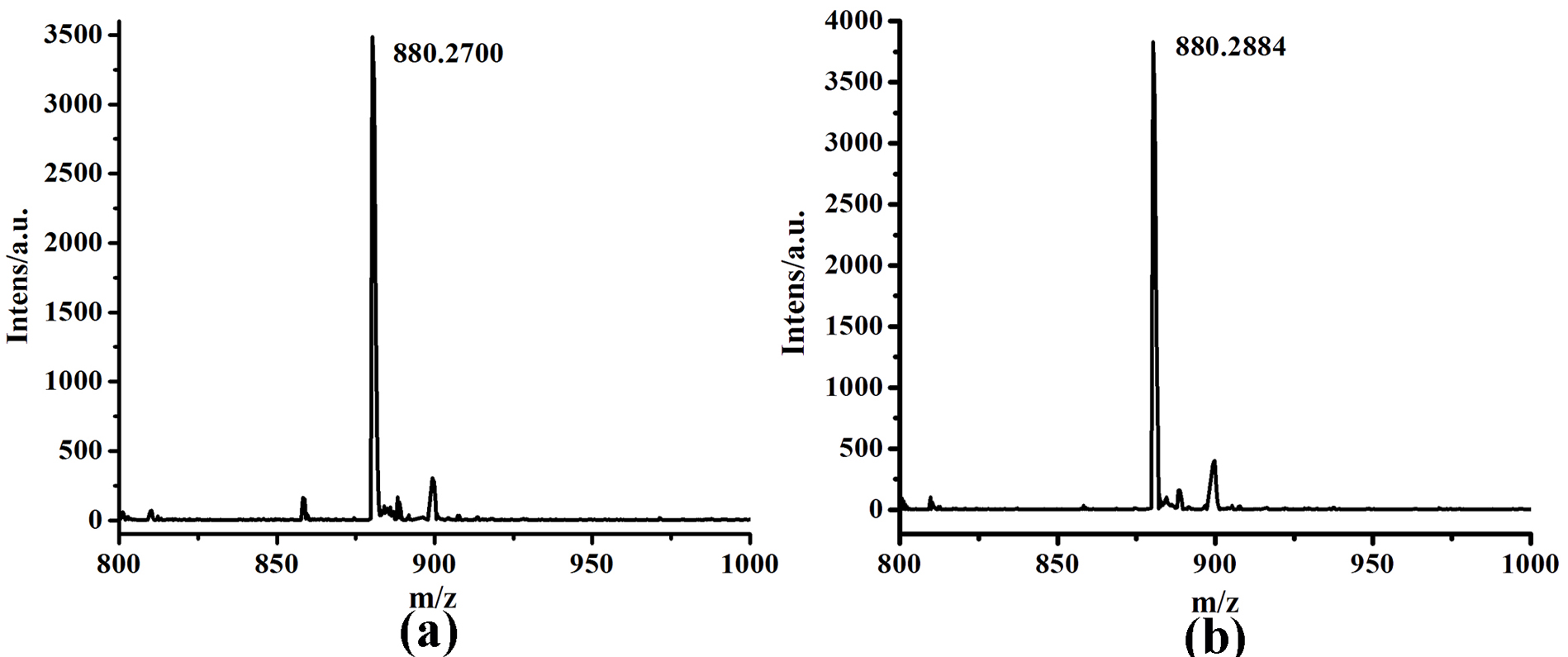
**

**Figure S4.** MALDI-TOF mass spectrum of homoternary complexes D-NA_2_@Q[8] (a) and L-NA_2_@Q[8] (b).


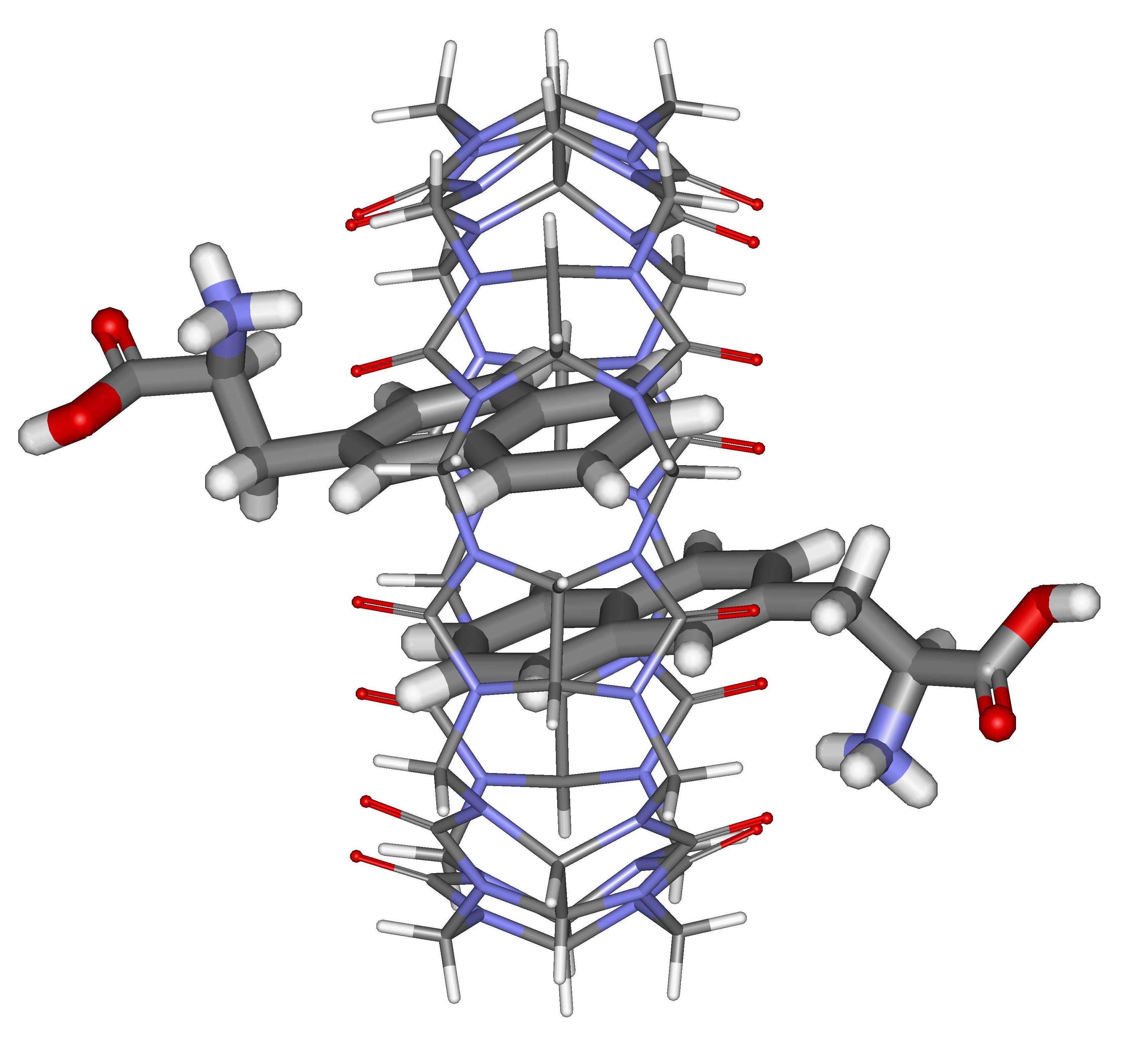


**Figure S5.** X-ray crystal structure of the homoternary complex L-NA_2_@Q[8]. Free L-NA molecules, solvate water molecules and [CdCl_4_]^2-^ anions are omitted for clarity.

**Table S1** Complex stability constant (*K*_a_), enthalpy (Δ*H*), and entropy changes (*T*Δ*S*) for D-NA_2_@Q[8] and L-NA_2_@Q[8].

| Experiment | D-NA_2_@Q[8] | L-NA_2_@Q[8] |
| --- | --- | --- |
| Model  *K*_a1_(1/M)  *K*_a2_(1/M)  Δ*H*_1_(kJ/mol)  Δ*H*_2_(kJ/mol)  Δ*S*_1_(J/mol)  Δ*S*_2_(J/mol) | Multiple Sites  (1.12±0.71)×10^6^  (5.81±0.27)×10^5^  -50.12±2.59  -6.17±2.56  -5.23  7.05 | Multiple Sites  (2.69±0.36)×10^6^  (1.18±0.15)×10^5^  -48.97±4.42  -3.97±4.77  -4.05  8.38 |
